# Supplementary material for: A Comprehensive Review of Recent Research into the Effects of Antimicrobial Peptides on Biofilms—January 2020 to September 2023
Source: Antibiotics (Basel). 2024 Apr 9;13(4):343. doi: 10.3390/antibiotics13040343 (PMC11047476; doi:10.3390/antibiotics13040343)
Supplement: Supplementary file 1 [file antibiotics-13-00343-s001.zip › antibiotics-2934978-supplementary.pdf]

Supplementary Table S1. Range of AMPs and sources referenced in this review article.

AMP Name	Origin	Additional Information	Number of Publications	Reference
AMP-17	AMP derived from *Musca domestica*	MLSTKQLVILGLLGCLSTLQA	2	**85, 86**
AMPR-11	AMP derived from Romo1 (reactive oxygen species modulator 1)	KTMMQSGGTFGTFMAIGMGIR	1	**102**
Ano-1ß	β-Ala modified-AMP analogue of anoplin (AMP from the venom of the solitary wasp *Anoplius samariensis*)	AβLLKRIKTLL	1	**11**
Ano-8ß	β-Ala modified-AMP analogue of anoplin (AMP from the venom of the solitary wasp *Anoplius samariensis*)	GLLKRIKAβLL	1	**11**
AP7121	AMP produced by *E. faecalis*	Sequence not shown in the available publication.	1	**70**
As-CATH4	Cathelicidin identified from Chinese alligator *Alligator sinensis*	RRGLFKKLRRKIKKGFKKIFKRLPPVGVGVSIPLAGRR	1	**138**
As-CATH5	Cathelicidin identified from chinese alligator *Alligator sinensis*	TRRKFWKKVLNGALKIAPFLLG	1	**138**
ASP-1	Synthetic AMP	RRWVRRVRRWVRRVVRVVRRWVRR	1	**133**
ASP-2	Synthetic AMP	RWWRWWRRWWRR	1	**133**
Bactenecin	AMP isolated from bovine neutrophils	RLCRIVVIRVCR	2	**137,139**
BCp12	Milk-derived AMP	YLGYLEQLLRLK	1	**64**
BiP_Aa_2	Synthetic AMP derived from Cnidarian moon jellyfish polyps *Aurelia aurita*	QLFCTKLSPT	1	**104**
BiP_Aa_5	Synthetic AMP derived from Cnidarian moon jellyfish polyps *Aurelia aurita*	VRNSVLFRRRRLVC	1	**104**
Block	Synthetic AMP	H-KKKKKKKKGGGLLALLALLA-NH2	1	**92**
BmKn-2 variants	Variants from AMP from scorpion (*Buthus martensii* Karsch) venom	FIGAIARLLSKIF	1	**94**
BPIFA1-derived AMP3	Synthetic AMP derived from BPI fold containing family member A1 (BPIFA1), a human natural defense protein	PIQGLLDSLTGILNKVLPHLVQGNVCPLVNHVLRGL	1	**80**
Brevinine-1E-OG9	AMP isolated from skin secretions of the frog *Odorrana grahami*	FPLIAGLAANFLPKILCKIARKC	1	**67**
G9c-De-NH2	Analogue of Brevinine-1E-OG9 (Frog skin-derived AMP)	FPLIARLAAKFLPKIL-NH2	1	**67**

C10-KR8d	Residues 18-29 of LL-37 (made of D-amino acids) linked to a C10 acyl chain	KRIWQRIK	1	**115**
CA(1-7)M(2-9)NH2	Cecropin A-melittin hybrid peptide	Sequence not shown in the available publication.	1	**72**
Capitellacin	AMP from marine polychaeta *Capitella teleta* (annelide worm)	SPRVCIRVCRNGVCYRRCWG	1	**107**
CD4-PP	Synthetic AMP made by dimerization and backbone cyclization of LL-37-derived peptide KR-12	AGGKRIVKRIKKFLRGAGGKRIVKRIKKFLRG	1	**117**
Cecropin A (CecA)	Synthetic AMP corresponding to a peptide from the greater wax moth *Galleria mellonella*	KWKIFKKIEKAGRNIRDGIIKAGPAVSVVGEAATIYKTG	4	**12, 90, 118, 142**
Ceragenin CSA-13	Synthetic AMPs derived from common bile acids	NOA	1	**118**
Ceragenin CSA-90	Synthetic AMPs derived from common bile acids	NOA	1	**118**
CGA-N9-C8	Analogue of AMP CGA-N9 derived from the N-terminus of human chromogranin A	N-octanoic-RILSILRHQ	1	**84**
Chol-37(F34-R)	Cholesterol-modified AMP PMAP-37(F34-R) derived from PMAP-37 from porcine myeloma cells	GLLSRLRDFLSDRGRRLGEKIERIGQKIKDLSERFQS	1	**42**
Citropin 1.1	AMP produced by both the dorsal and submental glands of the Australian green tree frog *Litoria citropa*	GLFDVIKKVASVIGGL-NH2	1	**72**
cLFchimera	Heterodimeric peptide designed to mimic two antimicrobial domains, Lactoferricin (LFcin) and Lactoferrampin (LFampin)	Sequence not shown in the available publication.	1	**97**
Cnt[15-34]	C-terminal part of the AMP Crotalicidin derived from rattlesnake venom	KKRLKKIFKKPMVIGVTIPF	1	**82**
Colistin (Polymyxin E)	AMP produced by *Bacillus polymyxa*	Sequence not shown in the available publication. Complicated structure involving multiple Dab (AP02204)*	1	**16, 48**
CRAMP	Mouse AMP	Sequence not shown in the available publication. 33 amino acids (AP00281)*	1	**57**
Crustin	AMP isolated from Green tiger shrimp *Penaeus semisulcatus*	Sequence not shown in the available publication. 83 amino acids (AP01555)*	1	**105**
D51-derivatives D51-P11G and D51-P11K	Derivatives of synthetic AMP D51	FLFRVASKVFGALIGKFKKK and FLFRVASKVFKALIGKFKKK	1	**93**

DASamP1 (database-screened antimicrobial peptide 1)	Synthetic AMP	FFGKVLKLIRKIF	2	**137,139**
D-CONGA-Q7	AMP variant of the synthetic AMP D-amino acid CONsensus with Glycine Absent (D-CONGA)	RRWARRQLAFAFRR	1	**113**
Defensin-1	Derived from exosomes from honeybee honey *Apis mellifera*	Sequence not shown in the available publication.<br>95 amino acids according to P17722 (UniProt)*	1	**76**
D-GL13K	Synthetic AMP derived from the human salivary protein BPIFA2	GKIIKLKASLKLL	1	**36**
DP7	In-silico designed synthetic peptide	VQWRIRVAVIRK	2	**61, 95**
DSPE-HnMc	Chimeric antimicrobial lipopeptide	NOA	1	**28**
E6	Synthetic AMP	RRWRIVVIRVRRC	1	**34**
ε-PL (EPL, Epsilon poly-L-Lysine)	Naturally occurring AMP (originally discovered as a secreted product in actinomyces)	Sequence not shown in the available publication.	1	**15**
Gaduscidin-1	Natural AMP derived from atlantic cod (*Gadus morhua*)	Sequence not shown in the available publication.	1	**60**
GA-KR12	Synthetic AMP produced by fusing gallic acid (GA) to KR12, a peptide derived from LL-37	Gallic acid-KRIVQRIKDFLR	1	**116**
Gallidermin	*Staphylococcus gallinarum*-derived lanthionine containing AMP (lantibiotic)	MEKFNNDFNLDVTVSKD DAKKADVKITSKSLCTPG CVTGLLMGCAGSSATCNC SVHVG	1	**47**
GH12	Synthetic AMP	GLLWHLLHHLLH	4	**14, 50, 120, 121, 145**
Halictine-2 analogue	synthetically prepared analogue of the natural AMP halictine-2 isolated from venom of the eusocial bee *Halictus sexcinctus*	GLWVKLLKKILK	1	**135**
Haloganan (Hgn)	Isoform of HG1, an AMP derived from halucidin	KWQNALLHHGLNCAKG VLK	1	**73**
HBD2 (human beta-defensin 2)	Natural human AMP	GIGDPVTCLKSGAIC2HPV FCPRRYKQIGTCGLPGTKC CKKP	2	**55, 56**
HBD3 (human beta-defensin 3)	Natural human AMP	GIINTLQKYYCRVRGGRC AVLSCLPKEEQIGKCSTRG RKCCRRKK	3	**55, 56, 74**
Hc-CATH	Cathelicidin Identified from the sea snake *Hydrophis cyanocinctus*	KFFKRLLKSVRRAVKKFRK KPRLIGLSTLL	1	**138**
HG-1	AMP derived from halocidin, a natural AMP in tunicates.	KWLNALLHHGLNCAKG VLA	1	**73**

Hill-Cec1	Synthetic AMPs derived from black soldier fly *Hermetia illucens*	SWWKKVFKPVEKLGQRVRDATIQGIGIAQQGANVLATVRGG	1	**103**
Hill-Cec10	Synthetic AMPs derived from black soldier fly *Hermetia illucens*	RWYKKIFKPVEKAVQRVRDATLQALGVAQQAANVYATAQGA	1	**103**
HNP-1	Member of the α-defensin family found in the human respiratory tract	ACYCRIPACIAGERRYGTCIYQGRLWAFCC	1	**49**
HNP-2	Members of the α-defensin family found in the human respiratory tract	CYCRIPACIAGERRYGTCIYQGRLWAFCC	1	**49**
Jelleine-1 analogue 15	Jelleine-1 was isolated from royal jelly of honeybees	PWRLRLRL	1	**59**
Jellein-3	Derived from exosomes from honeybee honey *Apis mellifera*	EPFKLSLHL	1	**76**
JIChis-2	Synthetic AMP variant of Jelleine-1 from the royal jelly of *Apis mellifera iberiensis*	PWRISIRLAAC	1	**38**
K11	Synthetic AMP inspired by natural AMPs (cecropin A, melittin and maganin 2)	KWKSFIKKLTKKFLHSAKKF	1	**16**
KP	Decapeptide derived from the sequence of the variable region of a single-chain recombinant anti-idiotypic antibody	AKVTMTCSAS	1	**51**
KWI18	Synthetic AMP based on *in silico* cleavage of trypsin inhibitor isolated from *Inga laurina* seeds (ILTI)	KWIRRIIRDYKKFFIKFI	1	**123**
L18R	Translated product of immunoglobulin gene J (locus heavy, IGHJ2	LLVLRSLGPWHPGHCLLR	2	**51, 134**
L5K5W	Synthetic amphipathic α-helical AMP	KKLWKWLKKLL	2	**137, 139**
LA-Bac8c	Synthetic AMP composed of lipoic acid (LA) linked to octapeptide Bac8c, a derivate from Bactenecin extracted from bovine neutrophils	lipoic acid (LA) or {5-(1,3-dithiolan-4-yl)pentanoic acid} linked to octapeptide Bac8c (RIWVVIWRR)	1	**114**
Lactoferrin (or Lactotransferrin)	Natural human AMP	Sequence not shown in the available publication. 710 amino acids according to O2788 (UniProt)*	1	**79**
Lactoferricin (LfcinH)	AMP derived from human lactoferrin through pepsin-mediated proteolysis	TKCFQWQRNMRKVR-G-PPVSCIKRDS	1	**100**
Laterosporulin	Natural AMP from soil bacteria *Brevibacillus* spp.	Sequence not shown in the available publication. 49 amino acids (AP01963)*	1	**39**
LF-1	Lactotransferrin-derived AMP	WKLLRKAWKLLRKA	1	**78**

Lf6-GG	Synthetic symmetric end AMP	RRWQWRGGRWQWRR	1	**110**
Lf6-pP	Synthetic symmetric end AMP	RRWQWRpPRWQWRR	1	**110**
LL-37	Natural human cathelicidin	LLGDFFRKSKEKIGKEFKRI VQRIKDFLRNLVPRTES	13	**13, 18, 45, 49, 62, 79, 89, 99, 100, 115, 117, 118, 129**
Lysozyme	Natural human AMP	Sequence not shown in the available publication. 130 amino acids (AP02257)*	3	**52, 79, 89**
MAA-41	Hybrid AMP of BMAP28 (a bovine antimicrobial peptide of the cathelicidin family) and LL-37	Sequence not shown in the available publication.	1	**129**
Magainin	AMP derived from the African clawed frog (*Xenopus laevis*)	Sequence not shown in the available publication, unclear if the publication refers to Magainin 1 or 2 - Magainin 1 GIGKFLHSAGKFGKAFVG EIMKS (AnaSpec)*; Magainin-2 GIGKFLHSAKKFGKAFVG EIMNS (AnaSpec)*	1	**118**
Mag2 (Magainin 2)	AMP derived from the skin of African clawed frog (*Xenopus laevis*)	GIGKFLHSAKKFGKAFVG EIMNS	1	**92**
Mag2-17base	AMP derivative from Magainin 2 from the African clawed frog (*Xenopus laevis*)	GIGKFLHSAKKFGKAFV	1	**92**
Melimine	Chimeric AMP from melittin (see table 1) and protamine (AAMP extracted from fish milt	TLISWIKNKRKQRPRVSRR RRRRGGRRRR	1	**58**
Melittin	Natural AMP derived from venom of the European honey bee *Apis mellifera*	GIGAVLKVLTTGLPALISW IKRKRQQ	1	**22**
MeIm-3.1-PP4	AMP derived from conjugation of methylimidazolium IL and synthetic AMP 3.1-PP4	Methylimidazolium IL coupled to the N-terminus of peptide 3.1-PP4 (KKLLKWLLKLLKTTKS)	1	**43**
MK58911-NH2 MK58911-NH2	Analogue from wasp peptide toxins	INWLKIAKKVKGML INWLHIAHHVHGML	1	**88**
MPX (mastoparan-X)	AMP isolated from wasp venom	INWKGIAAMAKKLL	2	**65, 96**
MRJP-1	Derived from exosomes from honeybee honey *Apis mellifera*	Sequence not shown in the available publication. 432 amino acids according to O18330 (UniProt)*	1	**76**
Nisin	Natural AMP (lantibiotic) from *Lactococcus lactis*	Sequence not shown in the available publication. 57	5	**15, 35, 46, 77, 93**

		amino acids according to P13068 (UniProt)*		
NK2A	Synthetic AMP based on bovine NK-lysin	TVIEVASKMCSKMRLLKG LCKSITKRFLRR	1	**92**
Octominin	Synthetic AMP based on a defense protein of *Octopus minor*	Sequence not shown in the available publication. GWLIRGAIHAGKAIHGLI HRRRH (DOI: 10.3390/md18010056)*	1	**20**
OctoPartenopin (P0)	Natural AMP extracted from suckers of *Octopus vulgaris*	AGTNK	1	**125**
Octopromycin	AMP derived from proline-rich protein 5 gene of *Octopus minor*	Sequence not shown in the available publication. RRLIRTDTGPIIYDYFKDQL LKKGMVILRESMKNLKG M (DOI: 10.1016/j.fsi.2021.07.01 9)*	1	**106**
OH-CATH-30	Fragment of OH-CATH missing 4 N-terminal amino acids, originally identified in king cobra (*Ophiophagus hannah*)	KFFKKLKNSVKKRAKKFF KKPRVIGVSIPF	1	**141**
P5	Synthetic AMP	RIVQRIKKWLLKWKKLGY	1	**136**
P6.2	Synthetic AMP	GLLRKWGKKWKEFLRRV WK	1	**136**
P12	Synthetic AMP	GWHHFFHFFHFF	1	**75**
PE2 26	Linear, fatty acid bearing AMP derivates of PE2 (AMP isolated from *Paenibacillus ehimensis* B7)	C7-Dab-Ile-Dab-d-Phe-Leu-Dab-d-Val-Leu-Tyr	1	**127**
PE2 27	Linear, fatty acid bearing AMP derivate of PE2 (AMP isolated from *Paenibacillus ehimensis* B7)	C8-Dab-Ile-Dab-d-Phe-Leu-Dab-d-Val-Leu-Tyr	1	**127**
Pep6	Synthetic AMP	RLRWWWRLR	1	**130**
Phormicin C-NS	AMP from housefly (*Musca domestica*) larval	Sorbic acid-ATCDLLSGTGVGHSACAA HCLLRGNRGG YCNGKGVCVCRN	1	**81**
Piscidin 1 (SBP1 and WBP1)	Synthetic AMP analogues of striped bass (*Morone saxatilis*) and white bass (*M. Chrysops*) piscidin 1, class I piscidin	Sequence not shown in the available publication. SBP1: MKCATLELVLSMVVLMA EPGDAFFHHIFRGIVHVG KTIHRLVTGGKAEQDQQD QQYQQEQQERRAQQYQR ENRERAAFD WBP1: MKCATLSLVLSMVVLMA EPGDAFFHHIFRGIVHVG KTIHKLVTGGKAEQDQQ DQQY2QDQQDQQAQQY QRENRERAAFD	1	**109**

		(DOI: 10.1371/journal.pone.0159423)*		
Piscidin 3 (SB/WBP3)	Synthetic AMP analogue of hybrid stripped / white bass (*M. Chrysops x M. saxatili*) piscidin 3, class I piscidin	Sequence not shown in the available publication. MRCITLELVLSMVVLMAE PGDAFIHHIERGIVHAGRS IGRELTGGKAQQER EQQDeREMDRERDAEN (DOI: 10.1371/journal.pone.0159423)*	1	**109**
Piscidin 4 (SBP4)	Synthetic AMP analogue of striped bass (*M. saxatilis*) piscidin 4, class II piscidin	Sequence not shown in the available publication. MKCVMIFLVLTLVVLMAE PGEGFERHLERGAKAIFRG ARQGWRAHKVVSRYRNR DVPETDNNQEEFYNQR (DOI: 10.1371/journal.pone.0159423)*	1	**109**
Piscidin 5 (WBP5	Synthetic AMP analogue of white bass *(M. Chrysops*) piscidin 5, class II piscidin	Sequence not shown in the available publication. MKCVMIFLVLTLVVLMAE PGEGLIGSLERGAKAIERG ARQGWRSHKAVSRYRAR YVRRPVIYYHRVYPNEER (DOI: 10.1371/journal.pone.0159423)*	1	**109**
PO3 (PEIg-PLO20)	Synthetic, star-shaped AMP with a PEI (polyethylenimine-g-poly (l-ornithine)) core and approximately eight surface primary amino groups	polyethylenimine-g-poly(l-ornithine) PEIg-PLO20	1	**112**
Polymyxin B	Natural AMP from *Bacillus polymyxa*	Sequence not shown in the available publication. N-[4-amino-1-[[1-[[4-amino-1-oxo-1-[[6,9,18-tris(2-aminoethyl)-15-benzyl-3-(1-hydroxyethyl)-12-(2-methylpropyl)-2,5,8,11,14,17,20-heptaoxo-1,4,7,10,13,16,19-heptazacyclotricos-21-yl]amino]butan-2-yl]amino]-3-hydroxy-1-oxobutan-2-yl]amino]-1-oxobutan-2-yl]-6-methyloctanamide-sulfuric acid (CAS registry number 1404-26-8)*	6	**11, 21, 48, 49, 52, 130**
Pom-1	AMP derived from Cuban freshwater snail (*Pomacea poeyana*)	Sequence not shown in the available publication.	1	**87**

		KCAGSIAWAIGSGLFGGA KLIKIKKYIAELGGLQ (DOI: 10.3390/pathogens100 40496)*		
Pom-2	AMP derived from Cuban freshwater snail (*Pomacea poeyana*)	Sequence not shown in the available publication. KEIERAGQRIRDAIISAAPA VETLAQAQKIIKGG (DOI: 10.3390/pathogens100 40496)*	1	**87**
Ponericin G1	AMP derived from venom of the predator ant *Pachycondyla goeldii*	NOA. GWKDWAKKAGGWLKKK GPGMAKAALKAAMQ (DOI: 10.1074/jbc.M1002162 00)*	1	**32**
PLL (Poly-L-Lysine)	Naturally occurring AMP	Sequence not shown in the available publication. (C6H14N2O2)x CAS registry number 25104-18-1*	1	**24**
Protamine	AMP isolated from salmon sperm (*Salmo salmine*)	MPRRRRSSSRPVRRRRRPR VSRRRRRRGGRRRR	1	**141**
Pt5-1c	Derivate of Pt5, an AMP derived from zebrafish (*Danio rerio*) phosvitin	Sequence not shown in the available publication. SRMKKWAKIIEKWRKWH KKRWLAHHSATK (DOI: 10.1016/j.fsi.2018.03.031)*	1	**17**
Q4-15-a1	Synthetic AMP derivative of GW-Q4	KKFATIAKKFINKLW	1	**53**
Random	Synthetic AMP	KKKLAKLKLGAKLKLKGK LGA	1	**92**
r(P)ApoBLPro	AMP derived from an antimicrobial region encrypted in the sequence of human apolipoprotein B	Sequence not shown in the available publication. PHVALKPGKLKFIIPSPKRP VKLLSGGNTLHLVSTTKT (DOI: 10.3390/ph14070631)*	1	**40**
RRP9W4N	Synthetic peptide derived from human proline arginine-rich end leucine-rich repeat protein (PRELP)	RRPRPRPRPWWWW	1	**37**
rScyreprocin	Recombinant AMP derived from natural AMP isolated from the gonads of mud crab *Scylla paramamosain*	MKEDSNILDKTAKMTKQ NKALLFTAGGAAAFMAG YYYYHCNYRNPAPKKSGS TTSQDKTDAQAVQSIPSPS GNKGKESKDPKVK	1	**126**
S6L3-33	Synthetic AMP	FKKFWKWFRRF	2	**137,139**
S100A12 (calgranulin C)	Natural human AMP	Sequence not shown in the available publication. 92 amino acids according to P80511 (UniProt)*	1	**54**

SAAP-148	Synthetic AMP	LKRVWKRVFKLLKRYWR QLKKPVR	1	**23**
SAMP-A4-C8	Synthetic AMP	n-octanoic-VRLLRRRI	1	**69**
SMAP-29	AMP derived from sheep cathelicidin	Sequence not shown in the available publication. RGLRRLGRKIAHGVKKYG PTVLRIIRIA (DOI: 10.1007/s00726-016-2170-y)*	1	**45**
Spa31	Truncated peptide derived from Spampcin, an AMP from Marine mud crab *Scylla paramamosain*	RRAAHGLLPRLRAPPPFH KRCVCLCRTAPPP	1	**132**
Sp-LECin	Truncated peptide derived from the mature peptide of SpCTL6, a c-type lectin homologue from the Marine mud crab *Scylla paramamosain*	GCVFLLPAKPHNYKKVFL SKGV	1	**124**
SS-BF-3	Synthetic AMP	NOA	1	**131**
Stripe	Synthetic AMP	KLLKKAGKLLKKAGKLLK KAG	1	**92**
SynPG-1	Hybrid AMP based on Protegrin-1, a porcine cathelicidin	GLRRLLRKIRGRWKGGGR GGRLCYCRRRFCVCVGR	1	**31**
TAT-RasGAP317-326	Chimeric AMP made of the cell-permeable HIV peptide TAT48-57 linked to a 10-amino acid sequence of the Src Homology 3 Domain (SH3 domain) of p120 RasGAP	RRRQRRKKRGGGDTRLNT VWMW	1	**122**
TC-19	Synthetic AMP derived from human thrombocidin-1-derived peptide L3	LRCMCIKWWSGKHPK	1	**101**
Temporin A	AMP derived from the European red frog *Rana temporaria*	FLPLIGRVLSGIL	1	**72**
Temporin G	AMP derived from the European red frog *Rana temporaria*	FFPVIGRILNGIL	1	**68**
TL (Temporin L)	AMP derived from the European red frog *Rana temporaria*	FVQWFSKFLGRIL	1	**19**
ToAP2	AMP derived from scorpion *Tityus obscurus* venom	FFGTLFKLGSKLIPGVMKL FSKKKER	1	**83**
TP4 peptide analogues, dN4 and dC4	Analogues from piscidin-like AMP TP4 of Nile tilapia, *Oreochromis niloticus*	TP4: FIHHIIGGLFSAGKAIHRLI RRRRR Sequence of analogues not shown in the available publication. dN4: IIGGLFSAGKAIHRLIRRRR R dC4: FIHHIIGGLFSAGKAIHRLI R	1	**91**

		(doi: 10.1371/journal.pone.0186442)*		
vCPP2319	Torque virus capsid protein-derived polycationic peptide	WRRRYRRWRRRRRWRRRPRR	1	**66**
W379	Synthetic AMP	Sequence not shown in the available publication. RRRWWWWV (DOI: 10.1002/adfm.202206936)*	2	**30, 33**
WLBU2	Synthetic AMP	RRWVRRVRRVWRRVVRVVRRWVRR	1	**111**
YS12	AMP derived from *Bacillus velezensis*	Sequence not shown in the available publication.	1	**128**
ZN-5	Synthetic Oreoch-2 analogue; Oreoch-2 (or TP4 piscidin) is an AMP derived from tilapia (*Oreochromis niloticus*)	FIIGGLFSAGKAIHRLIRRRRR	1	**108**
ZN-6	Synthetic Oreoch-2 analogue; Oreoch-2 (or TP4 piscidin) is an AMP derived from tilapia (*Oreochromis niloticus*)	FLFSAGKAIHRLIRRRRR	1	**108**
7e-SMAMP	Synthetic cationic amphipathic barbiturate	Guanidine barbiturate	1	**27**
15c	Synthetic AMP; quaternary ammonium iodide salt of a biphenylglyoxamide derivative	3-(2-(4′-Chloro-4-(octylsulfonamido)-[1,1′-biphenyl]-3-yl)-2-oxoacetamido)-N,N,N-trimethylpropan-1-aminium iodide	1	**119**
17base-Aib	AMP derivative from Magainin 2 from the African clawed frog (*Xenopus laevis*) with 2-aminoisobutyric acid (Aib) insertion	GIGKFLHSUKKFGKUFV	1	**92**
17base- Ac6c	AMP derivative from Magainin 2 from the African clawed frog (*Xenopus laevis*) with 2 1-aminocyclohexylcarboxylic acid (Ac6c) insertion	GIGKFLHSZKKFGKZFV	1	**92**
17base-Hybrid	AMP derivative from Magainin 2 from the African clawed frog (*Xenopus laevis*) with lysine insertion	GIKKFLKSZKKFVKZFK	1	**92**
1018-k6	*In silico* designed AMP	VRLIVLVRIWRR	1	**71**
1018M	Mutated version of the innate defense regulator peptide (IDR-)1018	VRLRWWRRRWRR	1	**63**
III5	Neolignan-AMP Mimic Conjugate	Sequence not shown in the available publication.	1	**41**
III15	Neolignan-AMP Mimic Conjugate	Sequence not shown in the available publication.	1	**41**

Not immediately available in Abstract or Publication	(Synthetic) AMPs without further information	Sequence not shown in the available publication.	5	**25, 26, 29, 44, 98**

NOA = No access to full publication (e.g., not an Open Access publication).

* = Extra information complete with its accompanying reference.
